# Serotonin facilitates late-associative plasticity via synaptic tagging/cross-tagging and capture at hippocampal CA2 synapses in male rats

**DOI:** 10.1093/oons/kvac002

**Published:** 2022-05-04

**Authors:** Amrita Benoy, Lik-Wei Wong, Niha Ather, Sreedharan Sajikumar

**Affiliations:** Department of Physiology, Yong Loo Lin School of Medicine, National University of Singapore, 117597 Singapore; Life Sciences Institute Neurobiology Programme, National University of Singapore, 117456 Singapore; Department of Physiology, Yong Loo Lin School of Medicine, National University of Singapore, 117597 Singapore; Life Sciences Institute Neurobiology Programme, National University of Singapore, 117456 Singapore; Healthy Longevity Translational Research Programme, Yong Loo Lin School of Medicine, National University of Singapore, 117456 Singapore; Department of Physiology, Yong Loo Lin School of Medicine, National University of Singapore, 117597 Singapore; Life Sciences Institute Neurobiology Programme, National University of Singapore, 117456 Singapore; Department of Physiology, Yong Loo Lin School of Medicine, National University of Singapore, 117597 Singapore; Life Sciences Institute Neurobiology Programme, National University of Singapore, 117456 Singapore; Healthy Longevity Translational Research Programme, Yong Loo Lin School of Medicine, National University of Singapore, 117456 Singapore

## Abstract

Synaptic plasticity in the hippocampal Cornu Ammonis (CA) subfield, CA2, is tightly regulated. However, CA2 receives projections from several extra-hippocampal modulatory nuclei that release modulators that could serve to fine-tune plasticity at CA2 synapses. Considering that there are afferent projections from the serotonergic median raphe to hippocampal CA2, we hypothesized that the neuromodulator serotonin (5-hydroxytryptamine; 5-HT) could modulate CA2 synaptic plasticity. Here, we show that bath-application of serotonin facilitates the persistence of long-term depression (LTD) at the CA3 Schaffer collateral inputs to CA2 neurons (SC-CA2) when coupled to a weak low frequency electrical stimulation, in acute rat hippocampal slices. The observed late-LTD at SC-CA2 synapses was protein synthesis- and N-methyl-D-aspartate receptor (NMDAR)-dependent. Moreover, this late-LTD at SC-CA2 synapses paves way for the associative persistence of transient forms of LTD as well as long-term potentiation to long-lasting late forms of plasticity through synaptic tagging and cross-tagging respectively, at the entorhinal cortical synapses of CA2. We further observe that the 5-HT-mediated persistence of activity-dependent LTD at SC-CA2 synapses is blocked in the presence of the brain-derived neurotrophic factor scavenger, TrkB/Fc.

## INTRODUCTION

The hippocampal subfield CA2, situated between the CA3 and CA1 subfields, acts as a small yet crucial connecting link and has emerged to be an important functional component of the hippocampal circuitry for information processing and memory [32, 39, 48]. CA2 neurons receive inputs from the Schaffer collateral (SC) fibers from CA3 at the proximal dendritic region and inputs from the entorhinal cortical layer II (EC LII) fibers at the distal dendritic region [6, 8, 25]. Parts of CA2 also receive monosynaptic inputs originating from the dentate gyrus [25]. Functionally, the CA2 subfield plays an important role in social memory [22] and social aggression [33, 46]. Anatomically, CA2 possesses a relatively high density of interneurons and an increased level of inhibitory transmission from interneurons [47]. CA2 neurons are also characterized by a distinctly higher expression of molecular markers such as regulator of G protein signalling 14 [31], higher endogenous calcium buffering capabilities and increased calcium extrusion rates [56], which among other factors contribute to the distinct patterns of synaptic plasticity expressed by CA2 pyramidal neurons compared with neurons of the CA1 subfield [5, 13]. One such distinction is the resistance to activity-dependent long-term potentiation (LTP) of synaptic transmission at the proximal CA3 SC inputs to CA2 neurons by typical LTP-inducing stimulation protocols [6, 64]. However, the distal entorhinal cortical (EC)→CA2 synapses inputs readily exhibit LTP in response to conventional stimulation protocols [6].

Interestingly, various neuromodulators serve to gate synaptic plasticity in CA2 neurons [4]. The present study aimed to elucidate the role of serotonergic neuromodulation in the regulation of plasticity at hippocampal CA2 synapses. Serotonergic afferents to the hippocampus arise from the raphe nuclei that send diffuse projections to all parts of the hippocampal formation [21]. In the rat hippocampus, it has been observed that the stratum oriens (SO) and stratum radiatum (SR) of the CA2 and CA3 areas, as also the stratum lacunosum moleculare, exhibit abundant serotonin-immunoreactive fibers [21, 23]. Although only few (or nearly absent) serotonergic fibers has been reported to be present in the stratum pyramidale and stratum lucidum of the CA3 area, a moderate number of serotonergic axons, mostly arising from the median raphe, has been observed to ramify within the CA2 stratum pyramidale and immediately beneath it, which is a distinct feature of the CA2 area in the rat hippocampus [21, 36]. Neuroanatomical tracing studies have also reported that the CA2 area receives projections from the serotonergic median raphe nucleus in the rat [58] and mouse hippocampus [8, 22]. Given these previous observations, we assumed that serotonin (5-hydroxytryptamine; 5-HT) could potentially modulate the plasticity of synaptic transmission at the SC and EC inputs in the CA2 SR and stratum lacunosum moleculare (SLM), respectively. We observed that serotonin facilitates the persistence of activity-dependent long-term depression (LTD) at SC synapses onto CA2 neurons (SC-CA2), which in turn enables the reinforcement of activity-dependent LTD via synaptic tagging as well as the reinforcement of activity-dependent LTP via synaptic cross-tagging, both at the EC synapses onto CA2 neurons (EC-CA2). Together, the results suggest that serotonin could potentially regulate CA2-dependent memory and behavior by modulation of these cellular correlates of learning and memory. We further elucidated a role for functional brain-derived neurotrophic factor (BDNF)/TrkB signaling in the observed 5-HT-mediated persistence of LTD at SC-CA2 synapses.

## MATERIALS AND METHODS

### Animals and preparation of hippocampal slices

Young adult postnatal day (P)35–49 male Wistar rats were used to prepare acute hippocampal slices for all experiments throughout this study. Animal procedures performed were approved by the guidelines of the Institutional Animal Care and Use Committee of the National University of Singapore. For the electrophysiology experiments, a total of 131 acute hippocampal slices (right/left hippocampi) were used from 65 male Wistar rats. The animals were purchased from In Vivos Pte Ltd (Singapore). All rats were kept in the institutional animal housing facility under 12-h light/dark cycle with food and water available *ad libitum*. Rats were anesthetized using CO_2_ and thereafter decapitated, following which the brain was isolated and quickly transferred to artificial cerebrospinal fluid (ACSF) maintained at 2°C–4°C and pH 7.2 to 7.4. The composition (in mM) of ACSF is as follows: 124 NaCl, 2.5 KCl, 2 MgCl_2_, 2 CaCl_2_, 1.25 NaH_2_PO_4_, 26 NaHCO_3_ and 17 D-glucose, equilibrated with 95% O_2_ and 5% CO_2_ (carbogen; flow rate 16 L/h) [9]. Using a manual tissue chopper, transverse hippocampal slices of 400-μm thickness were prepared from the isolated hippocampi (right/left). Slices were then placed on a nylon net in the interface chamber (Scientific Systems Design, Ontario, Canada) continuously perfused with carbogenated ACSF at a flow rate of 1 mL/min and maintained at 32°C. Slices were incubated for a 2–3-h recovery period prior to placing electrodes. Anesthetization, dissection and transfer of slices to the chamber were performed quickly and did not exceed an average duration of 5 min [55].

### Extracellular field potential recording

**Figure 1:**
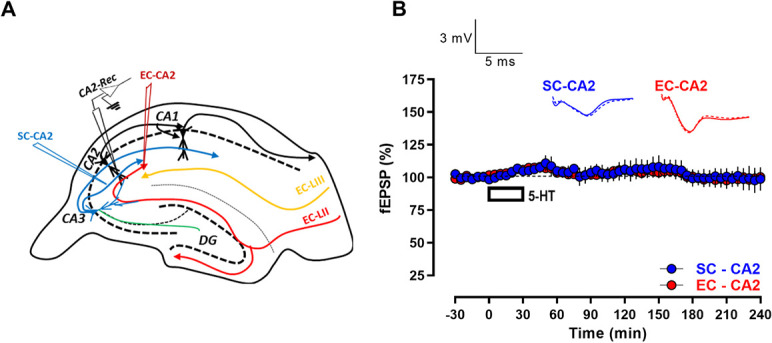
Bath-application of serotonin (5-HT) (10 μM) alone does not affect basal synaptic responses at EC-CA2 and SC-CA2 synapses. (A) A schematic diagram depicting the location of electrodes in acute hippocampal slices for the extracellular field electrophysiology experiments performed in this study. Blue inverted triangle represents the placement of electrode in the SR stimulating the CA3 SC afferents to CA2 neurons. Red inverted triangle represents the location of electrode in the stratum lacunosum moleculare stimulating the EC afferents to CA2 neurons. Black inverted triangle depicts the position of the recording electrode in the CA2 dendritic region. (B) Bath-application of 5-HT (10 μM) for 30 min following a 30 min stable baseline does not significantly alter synaptic responses at SC-CA2 and EC-CA2 synapses when both pathways are subjected to basal stimulation frequency throughout the recording period (*n* = 5). All data show mean ± SEM. Analog traces in (B) depict representative SC-CA2 (blue) and EC-CA2 (red) fEPSPs within the initial 30-min baseline recording before 5-HT application (closed line), and 60 min (dotted line) and 240 min (hatched line) after baseline. (Scale bars for analog traces in *B*: 3 mV/5 ms.)

Monopolar, lacquer-insulated stainless-steel electrodes (5 MΩ; AM Systems, Sequim, WA, USA) were used for stimulation and recording. Two stimulating electrodes, one stimulating the CA3 SC fibers (SC→CA2) and the other stimulating the EC fibers (EC LII→CA2), were positioned at the proximal (SR) and distal SLM CA2 dendritic regions, respectively, and field excitatory postsynaptic potentials (fEPSPs) upon stimulation of the proximal and distal inputs were recorded from the CA2 dendritic region (Fig. 1A). Identification of CA2 area and confirmation of SC→CA2 and EC LII→CA2 pathways were carried out using the pharmacological and electrophysiological methods described in our previous publication [9]. The input stimulation was generated and delivered using an isolated pulse stimulator (Model 2100; AM Systems, USA). The recorded signals obtained upon stimulation were amplified by a differential amplifier (Model 1700; AM Systems, USA), digitized using a CED 1401 analog-to-digital converter (Cambridge Electronic Design, Cambridge, UK) and monitored on-line with Intracell software (IfN, Magdeburg, Germany) where the initial slope function (millivolts per millisecond) of the fEPSP is measured for analysis. Afferent stimulation intensity vs. fEPSP slope (input–output relation) was plotted to determine the experiment stimulation strength, which was set to obtain an fEPSP response at 40% of the maximal fEPSP slope value. At this stimulus intensity, a stable baseline was recorded for 30 min before application of pharmacological agents or delivery of plasticity-inducing stimulation. The baseline stimulation current intensity so determined for each synaptic input was kept constant throughout the recording period. The pathway independence of the EC and SC synaptic inputs was confirmed using a cross-input paired-pulse facilitation protocol with an interstimulus interval of 50 ms similar to our previous study in which the absence of paired-pulse facilitation indicated that the two stimulating electrodes activated independent synaptic inputs [9].

The ‘weak’ low frequency stimulation (WLFS) consisted of 900 pulses at 1 Hz (pulse duration, 0.2 ms per polarity, a total of 900 stimuli). The ‘weak’ tetanization (WTET) protocol consisted of a single train of high frequency stimulation of 21 pulses at 100 Hz (pulse duration, 0.2 ms per polarity). The test stimulation consisted of four sweeps of 0.2 Hz biphasic constant current pulses (pulse duration, 0.1 ms per polarity) spaced at 5 s, at each recorded time point. The baseline and post-induction recordings (testing 1, 3 and 5 min post-WTET, or 1 and 5 min post-WLFS, and thereafter once every 5 min until the end of the recording period) were obtained with test stimulation. The test stimulation was set in the Intracell acquisition software to be automatically delivered every 5 min, and the average slope of the fEPSP responses generated by the four sweeps of each test stimulation was recorded as one repeat in the acquisition software.

### Pharmacology

Serotonin, 5-HT hydrochloride (H9523; Sigma-Aldrich), was freshly prepared before each application as a 10-mM stock solution in deionized water that was diluted to a 10-μM final working concentration in ACSF. The protein synthesis inhibitor, emetine dihydrochloride hydrate (emetine; E2375; Sigma-Aldrich) was prepared as a 20-mM stock solution in deionized water and stored at −20°C. The stock solution was diluted to a 20-μM final working concentration in ACSF before each application. The NMDAR antagonist, D-2-amino-5-phosphonovalerate (D-AP5; 0106; Tocris Bioscience) was stored at −20°C as a 50-mM stock solution prepared in deionized water that is diluted before application to a final working concentration of 50 μM in ACSF. The BDNF scavenger, recombinant human TrkB Fc chimera protein (TrkB/Fc; 688-TK; R&D Systems) was reconstituted in sterile phosphate-buffered saline and stored at −20°C. The stock solution was diluted in ACSF to a final concentration of 1 μg/mL before application. Light sensitive pharmacological substances were shielded from light during storage and bath-application. The duration and specific time points of application of individual pharmacological substances are described in the Results section.

### Data presentation and statistical analysis

The fEPSP values are represented as the mean of normalized fEPSP slope function (percentage of baseline) ± SEM. When comparing between two time points within the same group of data, the Wilcoxon signed-rank test (Wilcox test) was used for analysis. When comparing between two different groups of data at a specific time point, the values were analyzed using the Mann–Whitney *U* test. Nonparametric tests were used for the statistical analysis of the electrophysiological recordings as the sample size per series did not always guarantee a Gaussian normal distribution of the data. Differences at *P* < 0.05 were considered statistically significant. Prism (GraphPad Software, San Diego, CA) was used to plot the graphs and for the statistical analysis. Detailed description of statistical analysis for individual experiments is provided in the Results section.

**Figure 2:**
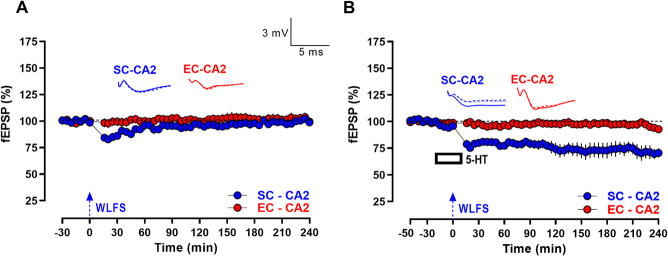
Serotonin facilitates persistence of activity-dependent LTD at SC-CA2 synapses. (A) Induction of short-lasting LTD (E-LTD) by weak low frequency stimulation (WLFS, 900 pulses at 1 Hz for a total of 15 min; indicated by a single hatched arrow) at the SC-CA2 synapses following a 30 min stable baseline recording, with SC-CA2 fEPSPs returning to baseline levels over time. The fEPSPs at EC-CA2 synapses remained at the baseline levels from the start of the experiment to the end indicating input specificity (*n* = 10). (B) L-LTD was observed at the SC-CA2 synapses when WLFS was delivered 20 min into bath-application of 5-HT (10 μM) for 30 min. The fEPSPs at EC-CA2 synapses subjected to basal stimulation frequency throughout remained at the baseline levels from the start of the experiment to the end indicating input specificity (*n* = 7). All data show mean ± SEM. Analog traces in (A and B) depict representative SC-CA2 (blue) and EC-CA2 (red) fEPSPs within 30 min before delivery of WLFS at SC-CA2 (closed line), and 60 min (dotted line) and 240 min (hatched line) post WLFS. (Scale bars for analog traces in A and B: 3 mV/5 ms).

## RESULTS

### Serotonin facilitates persistence of activity-dependent LTD at Schaffer collateral synaptic inputs to CA2 neurons

In order to understand whether serotonin (5-HT) alters synaptic efficacy at hippocampal CA2 synapses, it was first explored whether bath-application of 5-HT affects EPSPs at the SC-CA2 and EC-CA2 synaptic pathways at basal stimulation frequency (0.2 Hz) by performing field potential recordings in acute rat hippocampal slices. Schaffer collateral (SC) and entorhinal cortical (EC) afferents to CA2 form synapses at the proximal and distal CA2 dendritic regions, respectively, and the electrodes stimulating these fibers were appropriately positioned to allow independent stimulation of the two pathways while recording fEPSPs from the CA2 dendritic region (Fig. 1A). When 5-HT (10 μM) was bath-applied for 30 min following a 30-min stable baseline, fEPSPs at both EC-CA2 (Fig. 1B, red circles) and SC-CA2 (Fig. 1B, blue circles) synapses were not significantly different from their respective baseline values before application of 5-HT, at any given time point for the entire duration of the recording lasting up to 4 h post baseline (Fig. 1B, EC-CA2, 240 min: Wilcox test, *P* > 0.05; SC-CA2, 240 min: Wilcox test, *P* > 0.05). The concentration of 5-HT (10 μM) was adopted from previous studies examining the effect of 5-HT bath-application on cortical synaptic plasticity [15, 27].

Although this showed that 5-HT (10 μM) at basal stimulation frequency did not significantly alter excitatory postsynaptic responses at EC- and SC-CA2 pathways, it did not preclude the possibility that activation of serotonin receptors by 5-HT may have triggered cellular/synaptic changes that could be manifested under different synaptic stimulation paradigms but remained masked at basal stimulation frequency. Bath-application of 5-HT has been previously shown to block the induction of LTP by primed burst stimulation at the SC-CA1 pathway in rat hippocampal slices [7]. In the rat primary visual cortex, 5-HT (10 μM) has been shown to block the induction of LTP triggered by tetanic stimulation delivered during bath-application of 5-HT [15]. Based on these previous reports of possible inhibitory effects of 5-HT signalling on synaptic transmission, we hypothesized that 5-HT-mediated signalling could potentially serve to facilitate/enhance activity-dependent forms of LTD triggered by low frequency afferent stimulation at CA2 synapses. To explore this possibility, we first tested the effect of a weak low frequency stimulation (WLFS) delivered to the SC-CA2 synapses, which exhibited an early form of long-term depression (E-LTD) after a 30-min stable baseline recording (Fig. 2A), such that only a short-lasting depression ensues at SC-CA2 synapses following WLFS, that returns to baseline fEPSP levels by 80 min post WLFS (Fig. 2A, SC-CA2, blue circles, 80 min: Wilcox test, *P* > 0.05). The fEPSP levels of SC-CA2 synapses subjected to WLFS become comparable to the basal synaptic responses of control EC-CA2 pathway by 50 min post WLFS (Fig. 2A, 50 min: U-test, P > 0.05). Interestingly, it was observed that when 5-HT (10 μM) is bath-applied for a total duration of 30 min after the 30 min stable baseline recording, and WLFS (900 bursts at 1 Hz) delivered at the SC-CA2 pathway 20 min into 5-HT application, a long-lasting late-LTD (L-LTD) ensues at the SC-CA2 pathway (Fig. 2B, blue circles) with fEPSPs remaining significantly below baseline levels until the end of the recording period (Fig. 2B, SC-CA2, blue circles, 3 h post WLFS, Wilcox test, *P* = 0.0156; 4 h post WLFS, Wilcox test, *P* = 0.0156 and 180 min: *U*-test, *P* = 0.0175; 240 min: *U*-test, *P* = 0.0023). The EC-CA2 pathway was subjected to baseline stimulation throughout and displayed stable synaptic responses until the end of the recording period (Fig. 2A and B, EC-CA2, red circles, 240 min: Wilcox test, *P* > 0.05).

Taken together, the results demonstrated that application of serotonin transforms short-lasting activity-dependent E-LTD to persistent L-LTD at SC-CA2 synapses.

### Serotonin-mediated persistence of activity-dependent LTD at SC-CA2 synapses is dependent on NMDAR activation and protein synthesis

**Figure 3:**
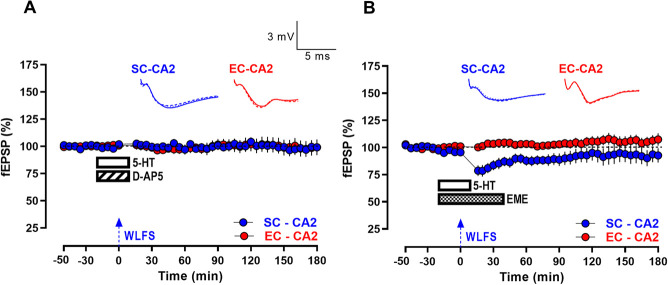
Serotonin-mediated persistence of WLFS-induced LTD at SC-CA2 synapses is NMDAR activation- and protein synthesis-dependent (A) E-LTD itself was absent at SC-CA2 following WLFS induction (900 pulses at 1 Hz for a total of 15 min; indicated by a single hatched arrow) at the SC-CA2 pathway delivered 20 min into the co-application of 5-HT (10 μM) and the NMDAR antagonist D-AP5 (50 μM) for 30 min (*n* = 7). (B) The reinforcement of E-LTD to L-LTD at SC-CA2 was absent such that only a short-lasting E-LTD was observed at SC-CA2 when WLFS was delivered at the SC-CA2 synapses 20 min into the co-application of 5-HT and the protein synthesis inhibitor emetine (EME; 20 μM) for 30 min, followed by application of emetine alone for another 30 min. The fEPSPs at EC-CA2 synapses remained at the baseline synaptic response levels from the start of the experiment to the end (*n* = 10). All data show mean ± SEM. Analog traces in (A and B) depict representative SC-CA2 (blue) and EC-CA2 (red) fEPSPs within 30 min before delivery of WLFS at SC-CA2 (closed line) and 60 min (dotted line) and 180 min (hatched line) post WLFS. (Scale bars for analog traces in A and B: 3 mV/5 ms.)

We next sought to explore the fundamental mechanistic underpinnings of the L-LTD expressed at SC-CA2 synapses following WLFS in the presence of 5-HT. Induction of activity-dependent forms of LTD in the hippocampal CA1 neurons has been previously established to be triggered by modest amounts of postsynaptic depolarization facilitated by NMDAR-dependent Ca^2+^ influx during repeated low frequency presynaptic stimulation [14, 35]. Consistent with this, the induction and therefore persistence of activity-dependent LTD facilitated by 5-HT at SC-CA2 synapses, was also observed to be NMDAR-dependent (Fig. 3A) as the co-application of 5-HT (10 μM) and the selective NMDA receptor antagonist D-AP5 (50 μM) abolished the acute as well as sustained depression in response to WLFS at SC-CA2 (Fig. 3A, SC-CA2, blue circles, 20 min: Wilcox test, *P* > 0.05; 180 min: Wilcox test, *P* > 0.05). The fEPSP values between SC-CA2 synapses subjected to WLFS, and EC-CA2 synapses at basal stimulation frequency were comparable throughout, when D-AP5 was co-applied with 5-HT (Fig. 3A, *U*-test, *P* > 0.05, at any given time point).

As 5-HT facilitated the transformation of activity-dependent E-LTD at SC-CA2 to a persistent L-LTD, it was tested whether the lasting form of LTD was maintained by virtue of *de novo* protein synthesis. Persistence of synaptic plasticity to its late forms has been attributed to new macromolecular synthesis of plasticity-related products (PRPs) [24], and L-LTD induced by strong low frequency afferent stimulation of the hippocampal SC-CA1 pathway has been shown to be dependent on *de novo* protein synthesis for its persistence [52]. When the protein synthesis inhibitor, emetine (20 μM) was bath-applied along with 5-HT (30 min together with and 30 min after 5-HT application) as shown in Fig. 3B, the late phase of LTD at SC-CA2 was blocked with fEPSPs returning to baseline levels by 95 min post WLFS at SC-CA2 (Fig. 3B, SC-CA2, blue circles, 95 min: Wilcox test, *P* > 0.05).

The NMDAR- and protein synthesis-dependency of persistent late forms of activity-dependent synaptic plasticity also fulfills the pre-requisites for the occurrence of late associative plasticity via synaptic tagging and capture-like processes mediated by heterosynaptic interactions between activated synapses along the neuronal dendritic tree [19, 45, 53]. The NMDAR-dependence (only the synapses whose NMDARs are activated by the presynaptically released glutamate during WLFS, display the observed plasticity) gives rise to the synapse-specificity [35] of the observed 5-HT-mediated LTD enhancement at the WLFS-triggered SC-CA2 synapses. The *de novo* protein synthesis triggered in response to the expression of 5-HT-facilitated L-LTD at SC-CA2 synapses makes available a pool of PRPs that could potentially support/modulate plasticity at stimulated independent neighboring synapses along the proximodistal dendritic axis of CA2 neurons. These possibilities were next explored.

### Serotonin-mediated persistence of activity-dependent LTD at SC-CA2 synapses facilitates captured L-LTD at EC-CA2 via synaptic tagging

**Figure 4:**
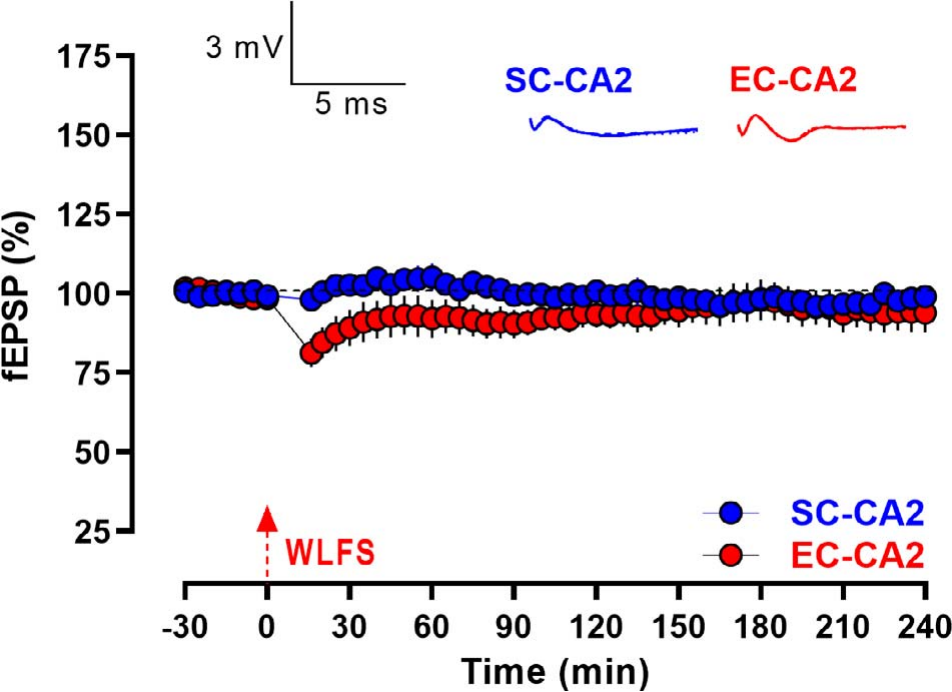
EC-CA2 synapses exhibit E-LTD in response to WLFS. E-LTD was observed following WLFS induction (900 pulses at 1 Hz for a total of 15 min; indicated by a single hatched arrow) at the EC-CA2 synapses following a 30-min stable baseline recording, with EC-CA2 fEPSPs returning to baseline levels over time. The fEPSPs at SC-CA2 synapses remained stable at the baseline levels from the start of the experiment to the end, indicating input specificity (*n* = 8). All data show mean ± SEM. Analog traces depict representative SC-CA2 (blue) and EC-CA2 (red) fEPSPs within 30 min before delivery of WLFS at EC-CA2 (closed line), and 60 min (dotted line) and 240 min (hatched line) post WLFS. (Scale bars for analog traces: 3 mV/5 ms.)

**Figure 5:**
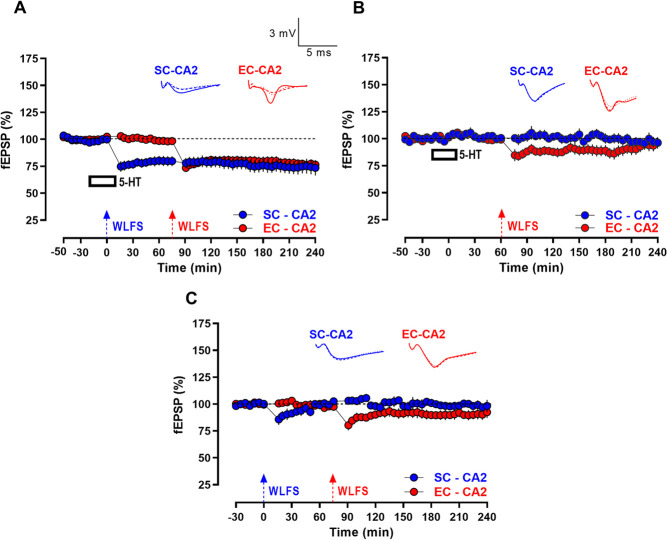
Serotonin-mediated L-LTD at SC-CA2 synapses engages in synaptic tagging and capture of L-LTD at EC-CA2 synapses. (A) A long-lasting L-LTD was observed following WLFS induction (900 pulses at 1 Hz for a total of 15 min; indicated by a single hatched arrow) at the SC-CA2 synapses, delivered 20 min into the bath application of 5-HT (10 μM) for 30 min after a 30-min stable baseline. This enables L-LTD to be exhibited, lasting until the end of the recording period, at the EC-CA2 synapses subjected to WLFS 1 h after the total duration of WLFS at SC-CA2 (*n* = 18). (B) 5-HT was bath-applied for 30 min following a 30-min stable baseline. WLFS was then delivered at EC-CA2 alone at the 60th minute, resulting in a short-lasting E-LTD at EC-CA2 with EC-CA2 fEPSPs returning to baseline levels over time. The fEPSPs at SC-CA2 synapses remained at baseline levels for the entire duration of the recording, indicating input specificity and the absence of any effect of 5-HT on basal synaptic responses at SC-CA2 at baseline stimulation frequency (*n* = 8). (C) After a 30-min stable baseline, WLFS was delivered to the SC-CA2 pathway, and 1 h after the total duration of WLFS at SC-CA2, the EC-CA2 synapses were subjected to WLFS, resulting in only a short-lasting E-LTD at EC-CA2 (*n* = 6). All data are presented as mean ± SEM. Analog traces for SC-CA2 (blue) fEPSPs in (A) and (C) depict representative SC-CA2 fEPSPs within 30 min before delivery of WLFS at SC-CA2 (closed line), and 60 min (dotted line) and 240 min (hatched line) post WLFS at SC-CA2. Analog traces for EC-CA2 (red) fEPSPs in (A) and (C) depict representative EC-CA2 fEPSPs within 30 min before delivery of WLFS at EC-CA2 (closed line), 60 min post WLFS at EC-CA2 (dotted line) and at the 240th min of the recording (hatched line). Analog traces for SC-CA2 (blue) fEPSPs in (B) depict representative SC-CA2 fEPSPs within the 30 min baseline before 5-HT application (closed line), 60 min into 5-HT application (dotted line) and at the 240th min of the recording (hatched line). Analog traces for EC-CA2 (red) fEPSPs in (B) depict representative EC-CA2 fEPSPs within 30 min before delivery of WLFS at EC-CA2 (closed line), 60 min post WLFS at EC-CA2 (dotted line) and at the 240th min of the recording (hatched line). (Scale bars for analog traces in A–C: 3 mV/5 ms.)

As 5-HT coupled to WLFS facilitates the persistence of LTD at SC-CA2 synapses to its late form in a protein synthesis-dependent manner as demonstrated in Fig. 3B, it was predicted that the PRPs synthesized in response to the L-LTD at SC-CA2 synapses could be captured by neighboring distal EC-CA2 synapses and potentially reinforce synaptic plasticity at weakly stimulated EC-CA2 synapses. EC inputs to CA2 neurons express only a transient early-LTD in response to WLFS delivered at the EC-CA2 pathway after a 30-min stable baseline recording, with fEPSPs at EC-CA2 synapses returning to baseline levels by 25-min post WLFS at EC-CA2 (Fig. 4, EC-CA2, red circles, 25 min: Wilcox test, *P* > 0.05). With the understanding that WLFS by itself is ineffective at triggering lasting LTD at EC-CA2 synapses, experiments were directed at understanding if the persistent L-LTD at SC-CA2 synapses facilitated by 5-HT could enable subsequently induced WLFS at EC-CA2 synapses to also express L-LTD potentially via synaptic capture (by LTD-specific tags set at EC-CA2 synapses subjected to WLFS) of LTD-specific PRPs synthesized from the signaling downstream of SC-CA2 L-LTD. Thus, WLFS was delivered to the EC-CA2 pathway 1 h following the total duration (900 bursts at 1 Hz, lasting a total of 15 min) of WLFS delivered to SC-CA2 during 5-HT application, as shown in Fig. 5A. As hypothesized, not only did 5-HT facilitate L-LTD at SC-CA2 synapses (Fig. 5A, SC-CA2, blue circles, 240 min: Wilcox test, *P* = 0.0019), it also enabled the persistence of LTD at EC-CA2 synapses for the remainder of the recording lasting until 165 min post WLFS delivered to the EC-CA2 pathway (Fig. 5A, EC-CA2, red circles; 240 min: Wilcox test, *P* < 0.0001). The control experiment in Fig. 5B showed that the observed persistence of LTD at EC-CA2 was absent when 5-HT was applied in the absence of WLFS at SC-CA2, resulting in EC-CA2 fEPSPs returning to its baseline levels by 50-min post WLFS at EC-CA2 (Fig. 5B, EC-CA2, red circles; 110 min: Wilcox test, *P* > 0.05). In a separate control experiment shown in Fig. 5C, it was also observed that in the absence of 5-HT, WLFS delivered at the EC-CA2 pathway 1 h following the total duration (900 bursts at 1 Hz, lasting a total of 15 min) of WLFS delivered at the SC-CA2 pathway was unable to facilitate L-LTD at both SC-CA2 and EC-CA2, such that fEPSPs at EC-CA2 synapses returned to its baseline levels by 45 min post WLFS at EC-CA2 (Fig. 5C, EC-CA2, red circles, 120 min: Wilcox test, *P* > 0.05). Interestingly, although WLFS induced a slight immediate depression after WLFS at the SC-CA2 pathway as shown in Fig. 5C, the difference with respect to its baseline was not statistically significant (Fig. 5C, SC-CA2, blue circles, 16 min: Wilcox test, *P* > 0.05), suggesting a heterogeneity in the expression of WLFS-induced E-LTD at SC-CA2 synapses under control conditions (compare against control experiment in Fig. 2A expressing a more pronounced E-LTD at SC-CA2 synapses in response to WLFS). Nevertheless, Fig. 5C demonstrates that WLFS alone at the SC-CA2 pathway in the absence of 5-HT, when preceding WLFS at the EC-CA2 pathway, does not suffice to reinforce WLFS-induced E-LTD to L-LTD at EC-CA2 synapses. Together, the experiments in Fig. 5A–C suggest that the transformation of E-LTD to L-LTD at the weakly stimulated EC-CA2 pathway is presumably mediated by the WLFS-induced setting of local LTD-specific synaptic tags at EC-CA2 synapses that capture LTD-specific PRPs synthesized in response to the preceding 5-HT-facilitated L-LTD expression at SC-CA2 synapses.

### Serotonin-mediated persistence of activity-dependent LTD at SC-CA2 synapses facilitates captured late-LTP at EC-CA2 via synaptic cross-tagging

**Figure 6:**
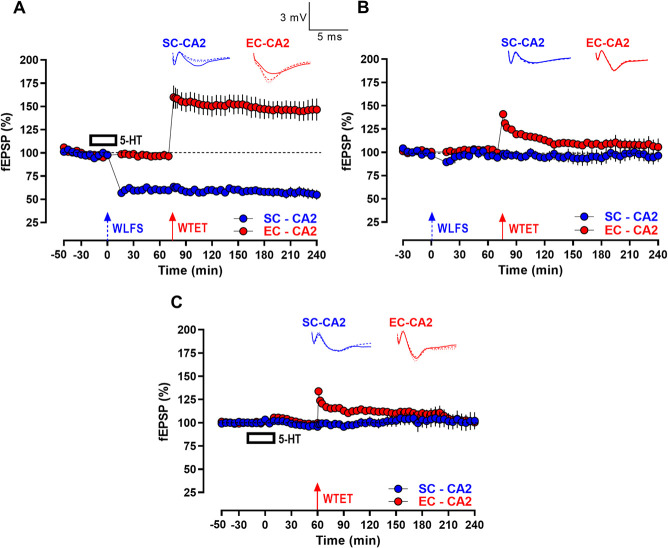
Serotonin-mediated L-LTD at SC-CA2 synapses facilitates transformation of E-LTP to L-LTP at EC-CA2 synapses via synaptic cross-tagging and capture processes. (A) L-LTD was observed following WLFS (900 pulses at 1 Hz for a total of 15 min; indicated by a single hatched arrow) at the SC-CA2 synapses, delivered 20 min into bath-application of 5-HT (10 μM) for 30 min. WTET (single train of 21 pulses at 100 Hz; indicated by a single solid arrow) was subsequently delivered at EC-CA2 1 h following the total duration of WLFS at SC-CA2, resulting in persistent expression of LTP at EC-CA2 synapses that lasted until the end of the recording period (*n* = 7). (B) After a 30 min stable baseline, WLFS was delivered to the SC-CA2 pathway, and 1 h after the total duration of WLFS at SC-CA2, the EC-CA2 synapses were subjected to WTET, resulting in only a short-lasting E-LTP at EC-CA2 with fEPSPs decaying to baseline levels over time(*n* = 6). (C) 5-HT was bath-applied for 30 min following a 30-min stable baseline. WTET was then delivered at EC-CA2 alone at the 60th minute, resulting in a short-lasting E-LTP at EC-CA2 with EC-CA2 fEPSPs returning to baseline levels over time. The fEPSPs at SC-CA2 synapses remained at baseline levels for the entire duration of the recording (*n* = 7). All data are presented as mean ± SEM. Analog traces for SC-CA2 (blue) fEPSPs in (A) and (B) depict representative SC-CA2 fEPSPs within 30 min before delivery of WLFS at SC-CA2 (closed line), and 60 min (dotted line) and 240 min (hatched line) post WLFS at SC-CA2. Analog traces for EC-CA2 (red) fEPSPs in (A) and (B) depict representative EC-CA2 fEPSPs within 30 min before delivery of WTET at EC-CA2 (closed line), 60 min post WTET (dotted line) and at the 240th min of the recording (hatched line). Analog traces for SC-CA2 (blue) fEPSPs in (C) depict representative SC-CA2 fEPSPs within the 30-min baseline before 5-HT application (closed line), 60 min into 5-HT application (dotted line) and at the 240th min of the recording (hatched line). Analog traces for EC-CA2 (red) fEPSPs in (C) depict representative EC-CA2 fEPSPs within 30 min before delivery of WTET at EC-CA2 (closed line), 60 min post WTET (dotted line) and at the 240th min of the recording (hatched line). (Scale bars for analog traces in A–C: 3 mV/5 ms.)

Late forms of plasticity, be it L-LTD or late-LTP (L-LTP), could lead to the synthesis of a pool of PRPs that presumably include process-specific PRPs for LTD and LTP, as well as process-unspecific, regulatory PRPs that may be common between LTD and LTP [18, 53, 24]. If this is the case, an L-LTD event at a given synaptic input can facilitate ‘captured’ L-LTP at weakly stimulated neighboring independent synaptic inputs if a reserve of LTP-specific PRPs and/or process-unspecific regulatory PRPs common between LTP and LTD, are synthesized in sufficient amounts in response to the L-LTD event, in addition to LTD-specific PRPs. This associative reinforcement of plasticity by heterosynaptic interactions between opposite plasticity events is suggested to be mediated by synaptic ‘cross-tagging and capture’ processes, wherein the early to late phase consolidation of the E-LTP/E-LTD event is facilitated by the synaptic capture of PRPs synthesized in response to the late forms of the opposite plasticity events, viz. L-LTD/L-LTP at neighboring synapses [53]. According to the ‘cross-tagging’ framework, the synaptic capture of process-relevant PRPs (process-specific and/or process-unspecific regulatory) is mediated by local process-specific synaptic tags generated at the activated synapses subjected to the opposite plasticity event-inducing stimuli [18, 53]. This scenario was tested in the case of 5-HT-facilitated protein synthesis-dependent L-LTD observed at SC-CA2 synapses. Thus, weak tetanic stimuli (WTET) consisting of a single train of high frequency stimulation (100 Hz) was delivered to the EC-CA2 pathway 1 h following the total duration of WLFS (900 bursts at 1 Hz, lasting a total of 15 min) delivered at the SC-CA2 pathway during 5-HT application, as shown in Fig. 6A. This resulted in the persistence of LTP following WTET at EC-CA2 synapses until the end of the recording period (Fig. 6A, EC-CA2, red circles, 240 min: Wilcox test, *P* = 0.0156). Consistent with the initial observations, L-LTD was also expressed at SC-CA2 synapses in response to WLFS delivered 20 min into the 5-HT application of 30 min after a 30 min stable baseline recording, such that SC-CA2 fEPSPs remained significantly depressed with respect to its baseline levels for up to 4 h post WLFS (Fig. 6A, SC-CA2, blue circles, 240 min: Wilcox text, *P* = 0.0156). The control experiment in Fig. 6B confirmed that the persistence of LTP at EC-CA2 was not observed when WTET was delivered at EC-CA2 synapses following WLFS at SC-CA2 in the absence of 5-HT, resulting in EC-CA2 fEPSPs returning to its baseline levels by 50 min post WTET (Fig. 6B, EC-CA2, red circles, 125 min: Wilcox test, *P* > 0.05). In the control experiment in Fig. 6B, it was also further confirmed that L-LTD was not observed at SC-CA2 in response to WLFS in the absence of 5-HT with fEPSPs at SC-CA2 synapses returning to baseline levels by 25 min post WLFS (Fig. 6B, SC-CA2, blue circles; 25 min: Wilcox test, *P* > 0.05). In a separate control experiment shown in Fig. 6C, it was demonstrated that 5-HT alone does not account for the observed persistence of WTET-triggered LTP at EC-CA2 in the absence of WLFS delivered to SC-CA2 during 5-HT application, such that the potentiation of fEPSPs following WTET at EC-CA2 synapses returned to its baseline levels by 115 min post WTET (Fig. 6C, EC-CA2, red circles, 175 min: Wilcox test, *P* > 0.05), while SC-CA2 synapses subjected to baseline stimulation throughout remained stable at basal synaptic response levels until the end of the recording period (Fig. 6C, SC-CA2, blue circles, 240 min: Wilcox test, *P* > 0.05). Together, the experiments in Fig. 6A–C establish that the LTP persistence observed at EC-CA2 synapses in response to WTET is mediated by heterosynaptic associativity with 5-HT-facilitated L-LTD at SC-CA2 synapses, presumably via synaptic ‘cross-tagging’ and capture processes.

**Figure 7:**
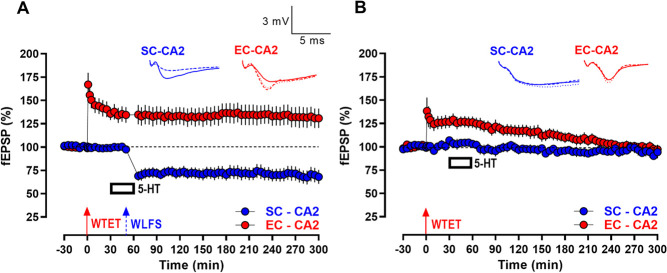
Reversing the order of delivery of the plasticity-inducing stimuli at SC-CA2 and EC-CA2 does not interfere with the expression of cross-captured L-LTP at EC-CA2 synapses facilitated by serotonin-mediated L-LTD at SC-CA2 synapses. (A) Following a 30-min stable baseline recording, WTET (single train of 21 pulses at 100 Hz; indicated by a single solid arrow) was delivered at EC-CA2 synapses. Thirty minutes following WTET at EC-CA2, 5-HT (10 μM) was bath-applied for 30 min, and WLFS (900 pulses at 1 Hz for a total of 15 min; indicated by a single hatched arrow) was delivered at SC-CA2 synapses 20 min into 5-HT application. This results in the expression of L-LTD at SC-CA2 synapses, as well the transformation of the WTET-induced E-LTP at EC-CA2 to L-LTP lasting until the end of the recording period (*n* = 10). (B) Following a 30-min stable baseline recording, WTET was delivered at EC-CA2 synapses. Thirty minutes following WTET, 5-HT was bath-applied for 30 min. This control experiment demonstrated that 5-HT alone, in the absence of WLFS delivered at SC-CA2 during 5-HT application, is insufficient to lead to persistent L-LTP at EC-CA2 post WTET as observed in (A), and EC-CA2 fEPSPs returned to baseline levels over time. SC-CA2 synapses were subjected to baseline stimulation frequency throughout and displayed stable basal synaptic responses for the duration of the recording (*n* = 7). All data are presented as mean ± SEM. Analog traces for SC-CA2 (blue) fEPSPs in (A) depict representative SC-CA2 fEPSPs within 30 min before delivery of WLFS at SC-CA2 (closed line), 60 min post WLFS (dotted line) and at the 300th min of the recording (hatched line). Analog traces for EC-CA2 (red) fEPSPs in (A) depict representative EC-CA2 fEPSPs within 30 min before delivery of WTET at EC-CA2 (closed line), at 66 min post WTET (dotted line) and at 300 min post WTET (hatched line). Analog traces for SC-CA2 (blue) and EC-CA2 (red) fEPSPs in (B) depict representative SC-CA2 and EC-CA2 fEPSPs within 30 min before delivery of WTET at EC-CA2 (closed line), 60 min post WTET (dotted line) and 300 min post WTET (hatched line). (Scale bars for analog traces in A and B: 3 mV/5 ms.)

If the persistence of LTP at EC-CA2 synapses observed in Fig. 6A was in fact mediated by a pool of process-relevant PRPs (LTP-specific and/or process-unspecific regulatory PRPs common to LTP and LTD) made available by the preceding 5-HT-facilitated L-LTD at SC-CA2 synapses, it would intuitively mean that the order of plasticity events should not affect the expression of captured L-LTP at EC-CA2 synapses so long as the PRPs are made available for synaptic capture before the decay of the process-specific synaptic tags. Therefore, even if 5-HT-facilitated L-LTD at SC-CA2 synapses were to be expressed after WTET is delivered at EC-CA2, it should still be possible for the EC-CA2 synapses to express persistent LTP by synaptic capture of PRPs synthesized by the succeeding L-LTD at SC-CA2 synapses. To test this possibility, we reversed the order of plasticity-inducing stimulation at SC and EC synaptic inputs in the cross-tagging experimental paradigm as shown in Fig. 7A such that WLFS during 5-HT application was delivered at SC-CA2 subsequent to WTET at EC-CA2 synapses (Fig. 7A). Confirming the proposed interchangeable nature of the cross-tagging and capture process, the reversed stimulation paradigm still resulted in WTET-induced E-LTP at EC-CA2 being transformed to L-LTP with EC-CA2 fEPSPs remaining significantly higher with respect to its baseline levels for up to 5 h post WTET at EC-CA2 (Fig. 7A, EC-CA2, red circles, 240 min: Wilcox test, *P* = 0.0039, 300 min: Wilcox test, *P* = 0.0098), while the succeeding 5-HT-facilitated L-LTD at SC-CA2 synapses lasted up to 250 min post WLFS at SC-CA2 (Fig. 7A, SC-CA2, blue circles, 240 min: Wilcox test, *P* = 0.0039, 300 min: Wilcox test, *P* = 0.0020). It was also confirmed that the E-LTP to L-LTP transformation at EC-CA2 synapses was not exhibited when, subsequent to WTET at EC-CA2, 5-HT was applied in the absence of WLFS at SC-CA2 (Fig. 7B), resulting in the synaptic potentiation at EC-CA2 returning to its baseline levels by 135 min post WTET (Fig. 7B, EC-CA2, red circles, 135 min: Wilcox test, *P* > 0.05), while SC-CA2 synapses subjected to baseline stimulation throughout exhibited stable basal synaptic responses until the end of the recording (Fig. 7B, SC-CA2, blue circles, 300 min: Wilcox test, *P* > 0.05). The experiments in Fig. 7A and B further reinstate that 5-HT-facilitated L-LTD at the proximal SC-CA2 synapses enables captured L-LTP at distal EC-CA2 synapses through synaptic cross-tagging and capture processes and confirm that the order of occurrence of the opposite plasticity events at SC- and EC-CA2 synapses are interchangeable for the expression of the captured L-LTP at EC-CA2 by synaptic cross-tagging.

### Inhibition of BDNF/TrkB signaling prevents serotonin-mediated late persistence of activity-dependent LTD at SC-CA2 synapses

**Figure 8:**
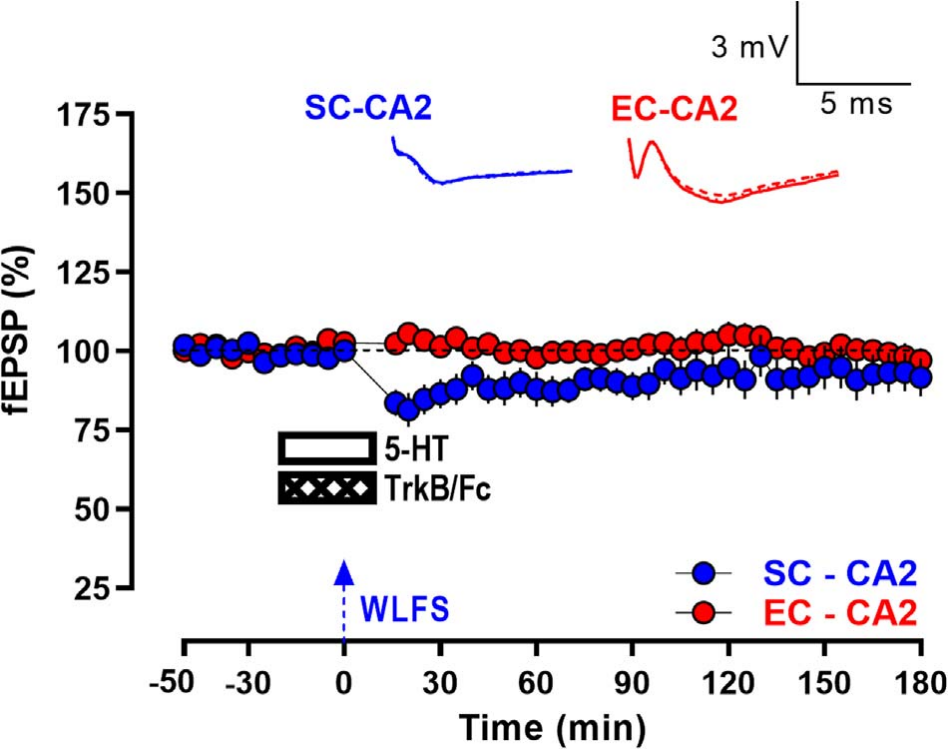
Inhibition of BDNF/TrkB signaling prevents serotonin-mediated late persistence of activity-dependent LTD at SC-CA2 synapses. After a 30-min stable baseline, the BDNF scavenger TrkB/Fc (1 μg/mL) was co-applied with 5-HT (10 μM) for 30 min, with WLFS (900 pulses at 1 Hz for a total duration of 15 min; indicated by a single hatched arrow) delivered to the SC-CA2 pathway 20 min into the drug application. This prevents the transformation of WLFS-induced E-LTD at SC-CA2 to L-LTD, such that SC-CA2 fEPSPs returned to baseline levels over time, while EC-CA2 fEPSPs subjected to baseline stimulation throughout exhibited stable basal synaptic response levels from the start of the experiment to the end (*n* = 15). All data are presented as mean ± SEM. Analog traces depict representative SC-CA2 (blue) and EC-CA2 (red) fEPSPs within 30 min before delivery of WLFS at SC-CA2 (closed line), and 60 min (dotted line) and 180 min (hatched line) post WLFS. (Scale bars for analog traces: 3 mV/5 ms.)

In a separate set of electrophysiology experiments to investigate the role of BDNF in the 5-HT-mediated persistence of activity-dependent LTD at SC-CA2 synapses, TrkB/Fc (1 μg/mL) that scavenges endogenously released BDNF was co-applied with 5-HT (10 μM) for a duration of 30 min following a 30 min stable baseline, and WLFS was delivered at the SC-CA2 pathway 20 min into the drug application (Fig. 8). Interestingly, TrkB/Fc when co-applied with 5-HT was able to block the late phase of LTD at SC-CA2 synapses, with the synaptic responses at SC-CA2 gradually returning to baseline levels by 75 min post WLFS (Fig. 8, SC-CA2, blue circles, 75 min: Wilcox test, *P* > 0.05), demonstrating the requirement of functional BDNF/TrkB signaling for the late maintenance of 5-HT-mediated activity-dependent LTD at SC-CA2 synapses.

## DISCUSSION

Serotonin (5-HT) is a monoamine modulatory neurotransmitter and widespread serotonergic innervation of the hippocampus arises from the midbrain raphe nuclei [21, 41]. Different results have been reported in previous studies exploring serotonergic modulation of synaptic plasticity in the hippocampus. 5-HT has been shown to facilitate potentiation of postsynaptic responses at CA3-CA1 synapses [40, 57]. In other studies, 5-HT has also been shown to inhibit LTP at commissural/associational (C/A)-CA3 synapses [59] and also in the CA1 region [7]. However, 5-HT-mediated modulation of synaptic plasticity in the CA2 region has not been previously explored. Using field electrophysiological recordings from the CA2 dendritic region, the present study reports that bath-application of 5-HT conjointly with WLFS facilitates the persistence of LTD at the SC-CA2 synapses. Postsynaptic inhibitory effects of 5-HT on hippocampal CA1 pyramidal neurons have been previously reported where 5-HT was shown to induce hyperpolarization by increasing potassium permeability [51]. Moreover, 5-HT has also been shown to induce LTD at corticostriatal synapses [37] and in the prefrontal cortex [65]. In the kitten visual cortex, LFS (1 Hz, 15 min) in the presence of 5-HT application has also been reported to facilitate LTD [26, 27]. Heterogeneity of activity-dependent LTD expression in CA2 neurons with low frequency stimulation (7.5 min stimulation at 2 Hz; −75 mV holding potential) has been previously observed in whole-cell voltage clamp recordings such that LTD was induced in some, but not all CA2 pyramidal neurons, unlike CA1 pyramidal neurons that express more robust LFS-induced LTD [64]. The present study shows that both the SC and EC inputs to CA2 neurons exhibit E-LTD in response to WLFS at the respective synaptic inputs under normal conditions. Application of 5-HT, however, transforms the WLFS-induced E-LTD at SC-CA2 synapses to L-LTD.

Further, we explored the mechanisms underlying this 5-HT-mediated persistence of LTD at SC-CA2 synapses and found it to be dependent on protein synthesis as the protein synthesis inhibitor, emetine, blocked the late phase of LTD in presence of 5-HT. It was also seen that the NMDA receptor antagonist, D-AP5, completely blocked the acute depression immediately after WLFS. This indicates that the post-synaptic calcium influx through NMDA receptors is crucial for activity-dependent LTD induction and the subsequent 5-HT-mediated facilitation of LTD persistence through protein synthesis. Thus, NMDA receptor activation is a necessary trigger for the activity-dependent induction of synaptic depression at SC-CA2 which paves way for further modulator-mediated reinforcement of the induced acute synaptic depression. BDNF is a key protein known to regulate hippocampal synaptic plasticity, including LTP and LTD, by activation of the cognate TrkB and p75 neurotrophin receptors that bind functionally active BDNF isoforms [28–30, 34, 61, 62]. A role for functional BDNF/TrkB signaling in the associative reinforcement of WLFS-triggered LTD by synaptic tagging/cross tagging and capture has been previously demonstrated at synapses in the hippocampal CA1 area [34, 54]. Consistent with these findings, we observed that the BDNF scavenger TrkB/Fc co-applied with 5-HT blocks the persistence of WLFS-triggered LTD at SC-CA2 synapses, demonstrating a role for functional BDNF/TrkB signaling in the 5-HT-facilitated reinforcement of LTD at SC-CA2.

The present study also crucially demonstrates that the 5-HT-facilitated L-LTD at SC-CA2 synapses enables the expression of late-associative plasticity at EC-CA2 synapses via heterosynaptic interactions involving synaptic tagging and cross-tagging. Although the molecular identity of synaptic tags is not definitively known, the results suggest that LTD-specific tags set at EC-CA2 synapses subjected to WLFS capture the LTD-specific and process-unspecific PRPs whose synthesis was triggered downstream of the 5-HT-facilitated long-lasting activity-dependent L-LTD at SC-CA2 that was shown to be protein synthesis-dependent. This synaptic tagging and capture process facilitates the expression of L-LTD at the weakly stimulated EC-CA2 synapses. Additionally, the present study also explored heterosynaptic interactions between opposite plasticity processes, viz. LTD at SC-CA2 and LTP at EC-CA2 synapses. WTET that normally elicits a short-lasting early-LTP (E-LTP) at EC-CA2 synapses was able to express a persistent L-LTP when the WTET at EC-CA2 was preceded or followed by WLFS at SC-CA2 during 5-HT bath-application. This suggests that the 5-HT-facilitated L-LTD at SC-CA2 synapses triggers the synthesis of a pool of PRPs, consisting of proteins that may be LTP-specific, LTD-specific as well as process-unspecific regulatory PRPs that may be common between LTD and LTP. The LTP-specific and process-unspecific PRPs from this pool captured by LTP-specific tags set at the weakly tetanized EC-CA2 synapses facilitates the late-associative reinforcement of E-LTP to L-LTP at EC-CA2. Thus, the opposite plasticity process, viz. LTP is reinforced at the distal EC-CA2 synapses through cross-tagging and capture of PRPs synthesized from the L-LTD at SC-CA2 synapses facilitated by 5-HT. Although heterosynaptic associativity of LTP has been reported previously at CA2 synapses by substance P modulation [9] and by inhibition of Group III metabotropic glutamate receptors [10], this study demonstrates CA2 heterosynaptic associativity triggered by LTD that facilitates the cellular consolidation of both LTD as well as LTP at neighboring synapses depending on the pattern of synaptic stimulation. Persistence of functional plasticity by synaptic tagging/cross-tagging and subsequent capture of PRPs, possibly enables consolidation of newly encoded memories into a lasting memory trace by virtue of its association with past and/or future strong synaptic events (strong memory/novelty) that upregulate the availability of PRPs [18, 50]. It could also be perceived as an efficient cellular mechanism for the compartmentalized storage of information at different synapses along the proximodistal dendritic axis of the neuron [18]. The results of the present study suggest that physiologically, such cellular memory consolidation processes in CA2 neurons are likely facilitated by serotonergic neuromodulation, although future *in vivo* studies that employ targeted stimulation of serotonergic terminals onto CA2 neurons are required to confirm this possibility. As the present study employed exogenous application of 5-HT to study its effects on synaptic efficacy, it is critical to perform further investigation to probe these effects under conditions of endogenous serotonin release. In addition, future studies should look in detail into the role of the specific 5-HT receptor subtypes involved in these effects. It is likely that Gi-coupled 5-HTRs or Gs-coupled 5-HTRs may be involved by their action on excitatory or inhibitory neurons.

Deficits in synaptic 5-HT levels by factors such as decreased 5-HT precursor synthesis, enhanced 5-HT-transporter function and the resultant greater synaptic 5-HT clearance, have been linked to psychiatric disorders such as depression [2, 60]. Our current findings are in similar line with previous studies showing the modulation of synaptic strength of CA2 pyramidal neurons via the neuropeptides vasopressin (Avp) and oxytocin (OT) [46]. Avp and OT are implicated in the control of social recognition memory, social behavior that includes maternal care and pair-bonding, anxiety and aggressive behavior [11, 16, 17]. Pagani *et al.* reported a crucial role of vasopressin modulation on CA2 pyramidal neurons via its action on Avpr1b receptors and provided an inter-relationship between modulation of synaptic plasticity of CA2 pyramidal neurons by vasopressin and aggressive behavior. They also compared the effects of Oxtr and Avpr1b agonists in regulating synaptic responses in CA2, CA3 and CA1 pyramidal neurons of the hippocampus. Similar to the response by Avpr1b agonists, oxytocin receptors (Oxtr) were able to potentiate excitatory post synaptic currents in CA2 pyramidal neurons and not in CA1 neurons [46]. Vasopressin is involved in anxiogenic behavior [42] while OT has a role in decreasing anxiety and inhibiting defensive behavior [1, 20, 38]. The balance between OT and Avp receptor expression, activation and the concomitant modulation of neurons has been proposed to regulate social behavior [20, 44]. As selective alterations to hippocampal CA2 are implicated in the pathophysiology of neuropsychiatric disorders that affect social behavior [3, 12, 49, 63], it is possible that serotonergic modulation of CA2 neurons has a contributory role in the regulation of social cognition and social behavior.

We have reported recently that plasticity and associativity show sex-specific differences [43]; the current study investigated only male rat hippocampal CA2 region and it will be useful to look into sex-specific differences in future studies to get a fuller understanding of the effects of 5-HT on CA2 plasticity in both the sexes. Additionally, future comprehensive immunohistochemical or *in situ* hybridization studies of all 5-HTR subtypes would also provide useful insights into how 5-HT receptor expression varies among the different hippocampal subfields and to understand its specific role in the CA2 region. However, the present investigation serves as a fundamental *in vitro* study that sheds light on the role of serotonin in CA2 functional plasticity and heterosynaptic associativity, and calls for future *in vivo* investigation of these effects on learning, memory and behavior.

## Supplementary Material

suppl_data_kvac002

## Data Availability

The data that support the findings of this study are available from the corresponding author upon reasonable request.
