# Supplementary material for: Serotonin facilitates late-associative plasticity via synaptic tagging/cross-tagging and capture at hippocampal CA2 synapses in male rats
Source: Oxf Open Neurosci. 2022 May 4;1:kvac002. doi: 10.1093/oons/kvac002 (PMC10913837; doi:10.1093/oons/kvac002)
Supplement: suppl_data_kvac002 [file suppl_data_kvac002.zip › kvac002_REVIEWER COMMENTS oxfnsc-2021-001.docx]

REVIEWER COMMENTS

OXFNSC-2021-001 Serotonin facilitates late-associative plasticity via synaptic tagging/cross-tagging and capture at male rat hippocampal CA2 synapses

Original submission

Reviewer 1

In the study entitled „Serotonin facilitates late-associative plasticity via….” the authors investigated in impressive detail the effects of serotonin on plasticity in the CA2 region. This is an important question, since the CA2 region has now been identified as an important region of the hippocampus playing a central role in social memory and aggression. Further, it has also been shown that this region differs in its ability to express plasticity from other hippocampal subregions. In sum, it is of interest to understand plasticity in this region better.

The authors have demonstrated great expertise in their electrophysiological recordings. It is of high quality and they recorded over up to 4 hours, which is impressive. They convincingly demonstrate that 5HT influences LTD-expression at SC-CA2 synapses and that synaptic tagging and capture influences LTD at the neighboring EC-CA2 synapses. They correlate changes in BDNF and Arc with these phenomena.

In spite of this overall positive evaluation, several issues require clarification/some additional work:
1. How did the authors identify the CA2 region? It is a small region and it may vary re its precise location depending on the level of the slice. This reviewer would have expected some kind of post-hoc analysis of electrode sites/staining of the CA2 region using available markers. The authors should explain why this was not necessary.
2. All animals were performed in male rats. The CA2 regions shows a sex-specific difference. The authors should make it clear that their recordings are from male rats and, thus, limited. 
3. The authors state (p. 3) that CA2 receives direct input from mossy fibers. This is true for part of CA2: There seems to be a part of the CA2 region with mossy fiber input (proximal) and a part without (distal). Again, post-hoc stainings would have helped. In the introduction the authors should state that “parts of CA2 receive monosynaptic inputs…”.
4. The authors investigated the role of NMDARs and protein synthesis but not of mGluR1 or mGluR5 in the context of LTD. They should comment on or test a possible involvement of mGluRs.
5. Did the authors investigate 5HTR? There are several subtypes and the blockers are commercially available. This experiment could also have helped to strengthen the link to BNDF. If they cannot perform this experiment, the authors should at least discuss which receptors are likely candidates.
Minor
6. The species and sex (male rats) should be mentioned in the title.
7. Controls should be shown first in the figures. E.g. Fig. 2 – switch A and B.

Reviewer 2

The study shows that application of 5HT in combination with low frequency stimulation of the SC-CA2 connection is sufficient to generate plasticity related proteins in the CA2 neurons that can both extend long-term depression at SC-CA2 synapses as well as EC-CA2 synapses, as well as extend LTP at EC-CA2 synapses. Interesting PRPs do not appear to be generated when low frequency stimulation of EC-CA2 synapses are linked to 5HT. Moreover 5HT has no effect on either synapse in the absence of LFS, and consistent with this NMDA receptor inhibitors that block the short and long-term depression block the setting of PRPs. Some biochemical changes are seen (Decreases in BDNF and increases in ARC) with LFS in combination with 5HT, but the significance of this is unclear (requirement for BDNF for the extended long-term depression at SC-CA2 synapses is not consistent with the decrease in BDNF being important). 

The data in the study are mostly robust and the interpretations are clear. There are some outstanding questions (below) that could be answered, but this is a fairly complete story, but I think they should at least be discussed.

1. CA2 is molecularly quite distinct from the rest of the hippocampus and thus, which 5HT receptors are expressed in CA2 specifically could be addressed (Allan Brain Atlas would provide this information). Pharmacology would be nice but is not required. 

2. Transmitter that activate Gi/Go (mGLUR) and Gq (substance P) have been characterized by this lab on these synapses and their effects seem distinct from 5HT, suggesting actions on Gs receptors (??). This should be discussed. 

3. The biochemistry (BDNF and ARC) are weak (n=3) and no 5HT alone group was done. The decrease in BDNF seems to go against the experiment where basal BDNF levels were required for the effect. If these are PRPs, then protein synthesis should be required for the changes. If these are included in the paper, these additional controls are needed, (5HT alone, protein synthesis inhibitors), but I do not believe that the results add substantially to the paper and they could also be removed. 

4. The discussion Is overly broad and should really focus on the actual results and the effects of other neuromodulators on these pathways. The work on substance P from this lab were shown, but effects of Vasopressn and Oxytocin should also be mentioned,

Decision letter

16-Dec-2021 

Dear Dr. Sreedharan, 

Manuscript ID OXFNSC-2021-001 entitled "Serotonin facilitates late-associative plasticity via synaptic tagging/cross-tagging and capture at hippocampal CA2 synapses" which you submitted to the Oxford Open Neuroscience, has been reviewed.  Reviewers are positively-disposed toward publication, but also suggest major revisions to your manuscript.  Therefore, I invite you to respond to the reviewers' comments and revise your manuscript. Their comments are included at the bottom of this letter. 

To revise your manuscript, log into https://mc.manuscriptcentral.com/oxfnsc and enter your Author Centre, where you will find your manuscript title listed under "Manuscripts with Decisions."  Under "Actions," click on "Create a Revision."  Your manuscript number has been appended to denote a revision. 

You may also click the below link to start the revision process (or continue the process if you have already started your revision) for your manuscript. If you use the below link you will not be required to login to ScholarOne Manuscripts. 

*** PLEASE NOTE: This is a two-step process. After clicking on the link, you will be directed to a webpage to confirm. *** 

https://mc.manuscriptcentral.com/oxfnsc?URL_MASK=ce34b628680a4afbbc41b66426152794 

You will be unable to make your revisions on the originally submitted version of the manuscript.  Instead, revise your manuscript using a word program and save it on your computer.  Please also highlight the changes to your manuscript within the document by using the track changes mode in MS Word or by using bold or colored text. 

Once the revised manuscript is prepared, you can upload it and submit it through your Author Centre. 

When submitting your revised manuscript, you will be able to respond to the comments made by the reviewer(s) in the space provided.  You can use this space to document any changes you make to the original manuscript.  In order to expedite the processing of the revised manuscript, please be as specific as possible in your response to the reviewers. 

IMPORTANT:  Your original files are available to you when you upload your revised manuscript.  Please delete any redundant files before completing the submission. 

Because we are trying to facilitate timely publication of manuscripts submitted to the Oxford Open Neuroscience, your revised manuscript should be uploaded as soon as possible.  If it is not possible for you to submit your revision in a reasonable amount of time, we may have to consider your paper as a new submission. 

Thank you for submitting your manuscript to the Oxford Open Neuroscience and I look forward to receiving your revision. 

Sincerely, 
Dr. Alicia Izquierdo 
Senior Editor, Oxford Open Neuroscience 
aizquie@psych.ucla.edu 

Author response – has been archived

Revised submission

Reviewer 1

The authors have addressed my concerns.

Reviewer 2

My concerns have been addressed. I have no more comments.

Decision letter

02-Feb-2022 

Dear Dr. Sreedharan, 

It is a pleasure to accept your revised manuscript entitled "Serotonin facilitates late-associative plasticity via synaptic tagging/cross-tagging and capture at male rat hippocampal CA2 synapses" in its current form for publication in the Oxford Open Neuroscience. The comments of the reviewer(s) who reviewed your manuscript are included at the foot of this letter. 

Thank you for your contribution. On behalf of the Editors of the Oxford Open Neuroscience, we look forward to your continued contributions to the Journal. 

Sincerely, 
Dr. Alicia Izquierdo 
Senior Editor, Oxford Open Neuroscience 
aizquie@psych.ucla.edu
